# 45-Year Trends in Women’s Use of Time and Household Management Energy Expenditure

**DOI:** 10.1371/journal.pone.0056620

**Published:** 2013-02-20

**Authors:** Edward Archer, Robin P. Shook, Diana M. Thomas, Timothy S. Church, Peter T. Katzmarzyk, James R. Hébert, Kerry L. McIver, Gregory A. Hand, Carl J. Lavie, Steven N. Blair

**Affiliations:** 1 Department of Exercise Science, Arnold School of Public Health, University of South Carolina, Columbia, South Carolina, United States of America; 2 Center for Quantitative Obesity Research, Montclair State University, Montclair, New Jersey, United States of America; 3 Pennington Biomedical Research Center, Baton Rouge, Louisiana, United States of America; 4 Department of Epidemiology and Biostatistics, University of South Carolina, Columbia, South Carolina, United States of America; 5 Cancer Prevention and Control Program, University of South Carolina, Columbia, South Carolina, United States of America; 6 Ochsner Heart and Vascular Institute, The University of Queensland School of Medicine, New Orleans, Louisiana, United States of America; NIDDK/NIH, United States of America

## Abstract

**Context:**

Relationships between socio-environmental factors and obesity are poorly understood due to a dearth of longitudinal population-level research. The objective of this analysis was to examine 45-year trends in time-use, household management (HM) and energy expenditure in women.

**Design and Participants:**

Using national time-use data from women 19–64 years of age, we quantified time allocation and household management energy expenditure (HMEE) from 1965 to 2010. HM was defined as the sum of time spent in food preparation, post-meal cleaning activities (e.g., dish-washing), clothing maintenance (e.g., laundry), and general housework. HMEE was calculated using body weights from national surveys and metabolic equivalents.

**Results:**

The time allocated to HM by women (19–64 yrs) decreased from 25.7 hr/week in 1965 to 13.3 hr/week in 2010 (*P*<0.001), with non-employed women decreasing by 16.6 hr/week and employed women by 6.7 hr/week (*P*<0.001). HMEE for non-employed women decreased 42% from 25.1 Mj/week (6004 kilocalories per week) in 1965 to 14.6 Mj/week (3486 kcal/week) in 2010, a decrement of 10.5 Mj/week or 1.5 Mj/day (2518 kcal/week; 360 kcal/day) (*P*<0.001), whereas employed women demonstrated a 30% decrement of 3.9 Mj/week, 0.55 Mj/day (923 kcal/week, 132 kcal/day) (*P*<0.001). The time women spent in screen-based media use increased from 8.3 hr/week in 1965 to 16.5 hr/week in 2010 (*P*<0.001), with non-employed women increasing 9.6 hr/week and employed women 7.5 hr/week (*P*<0.001).

**Conclusions:**

From 1965 to 2010, there was a large and significant decrease in the time allocated to HM. By 2010, women allocated 25% more time to screen-based media use than HM (i.e., cooking, cleaning, and laundry combined). The reallocation of time from active pursuits (i.e., housework) to sedentary pastimes (e.g., watching TV) has important health consequences. These results suggest that the decrement in HMEE may have contributed to the increasing prevalence of obesity in women during the last five decades.

## Introduction

Despite the significant mortality, morbidity and economic burden engendered by the recent increase in the prevalence of obesity and non-communicable chronic diseases [1], [2], there are few investigations of longitudinal population-level data that allow examination of trends in presumptive risk factors.

### Etiologies of Obesity and Energy Balance

The origins of the obesity epidemic are in dispute [3]. Nevertheless, it is widely accepted that increments in body weight and adiposity, at the most fundamental level, are the result of chronic positive energy balance (i.e., energy expenditure [EE]<energy intake [EI]) [4]. While this imbalance may be engendered via decrements in EE (e.g., diminished physical activity) and/or increments in EI, the imprecision of current methods for measuring population-level EI [5], [6], [7] and EE [8] limits the accurate quantification of the energy balance equation. Therefore, data on population-level trends in risk factors (e.g., physical activity, PA) [9] may provide essential contextual evidence by which to inform public policy.

#### Energy intake and obesity

Given the lack of reliable data on population-level EI [5], [6], [7], recent publications have suggested that superficial food economics (e.g., alterations in food supply or food costs) are responsible for the obesity epidemic [10], [11]. This unidimensional determinism (i.e., economic forces cause obesity) is implausible given two well-established facts. First, humans adapt physiologically and behaviorally to perturbations in energy balance via an array of compensatory homeostatic mechanisms [12], [13], [14], [15], [16] and second, while food supply forces (e.g., availability, price) affect purchase and perhaps utilization and waste, the mere presence of food and over-consumption of food are simply necessary but not sufficient conditions for long-term changes in adiposity [13], [15], [17], [18], [19], [20], [21]. This is most clearly demonstrated in countries in which the food energy supply increased [21], [22], [23] while BMI levels were stable or decreasing [22], [24], [25], and the converse context in early 20^th^ century US in which the food supply was decreasing [26] while BMI was increasing [27].

Given the homeostatic nature of human behavior and energy physiology, and the fact that physical activity and body composition determine nutrient partitioning (i.e., the metabolic fate of the food we consume), we postulate that it is highly unlikely that the obesity epidemic is the result of superficial food economics.

#### Data on population-level trends

The complex, multi-dimensional nature of the obesity epidemic and lack of valid data on population trends in EI [5], [6], [7] necessitates examinations of trends in population-level risk factors if public health policy is to be informed by the best available science. Recent publications have concluded that PA has not declined over the recent past despite two major limitations. First, these studies focused on highly selected, non-representative samples and used indirect, inherently confounded measures of PA such as the physical activity level (PAL) index (which conflates all non-resting EE with PA) [28]. Second, most previous empirical work examined domains of PA that account for only a small portion of total EE [29].

In 2011, Church et al., examined occupational EE via US Bureau of Labor Statistics” surveys that included data on employment and earnings from more than 140,000 businesses, government agencies and >440,000 individual worksites, and found a significant decreasing trend over the past 50 years. They concluded that the reduction in this single domain of PA may account for a considerable portion of the increase in mean body weights for men and women [30]. The present study sought to complement Church et al.”s results by providing longitudinal data on another domain of PA: household management (HM). Since Church et al.”s work on occupational EE explained more of the increase in weight for men than women, and nearly all women (but not the majority of men) perform HM activities on a daily basis [31], we posited that a greater portion of women”s EE may be from HM. To test the hypothesis that trends in the time allocated to HM may contribute to longitudinal decrements in EE and potentially to gains in weight or adiposity, we examined nationally representative time-use diary data sources to analyze 45-year trends in the allocation of time, HM activities (e.g., meal preparation, laundry, and cleaning) and energy expenditure in women.

## Methods

### The Allocation of Time

Data on women”s” allocation of time were derived from nationally representative time-use diary data from 1965–2010. Time-use data have been demonstrated to be more reliable and valid for non-occupational PA than other traditional surveillance systems [32]. Importantly, this data source provides details that allow an examination of the reallocation of time between activities (i.e., activity displacement). For example, if individuals spend less time cooking and cleaning, they may spend more time exercising, sleeping, or watching television TV.

The American Heritage Time Use Study (AHTUS) [33] database was the source of nationally representative historical time-use data, and provided harmonized data on specific activities relevant to HM. The AHTUS dataset was produced specifically to analyze trends in total work output (i.e., paid and unpaid work) [34]. The harmonization process standardized multiple sources of data into a unified context for the purpose of comparative analyses. This process is complex and detailed information is provided by Fisher et al. (2011) [33] and Egerton et al. (2005) [34]. The AHTUS datasets consist of >50,000 diary days from 1965–2010. The number of weighted diaries from women (age 19–64 years) available for analysis were: 1036 for 1960s, 1924 for 1970s, 1420 for 1980s, 4117 for 1990s, 17,885 for 2003–2005, and 23,900 for 2006–2010.

Time-use data sources were divided into four areas: 1) paid work, 2) household (e.g., unpaid housework and family care), 3) personal care (e.g., grooming), and 4) free time (e.g., TV viewing, exercise). Time-use data were analyzed for the change in the amount of time spent in each area by examining >90 subcategories of daily activity. Our final analyses included only those activities that significantly affected total daily EE over the 45-year period. For this investigation, household management activities were comprised of the aggregate time spent preparing food (e.g., cooking, washing dishes), general cleaning (e.g., vacuuming), clothing maintenance (i.e., laundry) but not general child or adult care, vehicle or house maintenance (e.g., painting), gardening or lawn care. Screen-based media use was defined as the non-occupational use of television and computer during free time, and leisure time physical activity (LTPA) was defined as sport and exercise participation.

#### Employment status and age

Employment impacts the time allocated to HM [31], [35], [36]. For this study, the terms “employed” and “non-employed” encompassed all women and referred to the respondent”s self-reported employment status based on paid work hours per week. Full time employment was >21 hr of paid work per week for 1965–1990 and >35 hr/week for 1990–2010. Age also impacts HM activities; therefore, the sample was divided into age groups (i.e., <35 years, 35–50 years, >50 years old).

#### Energy expenditure associated with activity

HM consists of numerous tasks of varying intensity and EE. The data used in this study did not have the detail necessary to delineate between the various components of HM. As such, a conservative Metabolic Equivalents (MET) value of 2.8 was assigned to HM activities. This value represents the EE per unit of time and was based on the Food and Agriculture Organization of the United Nations, World Health Organization and United Nations University (FAO/WHO/UNU) report on human food energy requirements [37], [38] and the 2011 Compendium of Physical Activities [39]. Walking (2–4 METs) accounted for the vast majority of LTPA in the AHTUS and US population [40], [41], [42]. Nevertheless, a liberal MET value of 4.5 was used for all LTPA to account for the infrequent occurrence of activities of higher intensity.

#### Energy expenditure and body mass

Increased body mass increases EE at rest and during PA. Since women were heavier in 2010 than in 1965, increments in the body weight used for the estimation of the HMEE and LTPA for each epoch were necessary. These increments were calculated from national surveys (NHES:1960–62; NHANES: 1971–74, 1976–80, 1988–94, 1999–2002 and 2003–2010) for the age group 19 to 64 [43]. The NHANES provides a representative sample of the civilian, non-institutionalized U.S. population via a complex, probability sampling design. The estimated body weights used for each epoch were 1960s = 65 kg; 1970s = 66 kg; 1980s = 69 kg; 1990s = 71 kg; 2005 = 74 kg; 2010 = 75 kg.

As per previous research on PA [30] and the 2011 Compendium [39], the estimated EE for each activity and time period was calculated from the equation: EE = (hours in activity per week x MET value for activity x mean body weight).

### Statistical Analyses

Data processing and statistical analyses were performed using SPSS V·19 in 2012. Decade-to-decade contrasts and trend analyses were conducted for HMEE and the allocation of time to HM, screen-based media, LTPA. Analyses accounted for the survey design via the incorporation of weighting to maintain a nationally representative sample. All analyses included adjusted means, and *p*<0.05 (2-tailed) indicated statistical significance.

## Results

Descriptions of the AHTUS population, have been published previously [33], [34]. The AHTUS survey distribution did not differ substantively from population statistics with the exception of a greater proportion of the respondents were of higher socio-economic status.

### Time Allocated to HM

**Figure 1:**
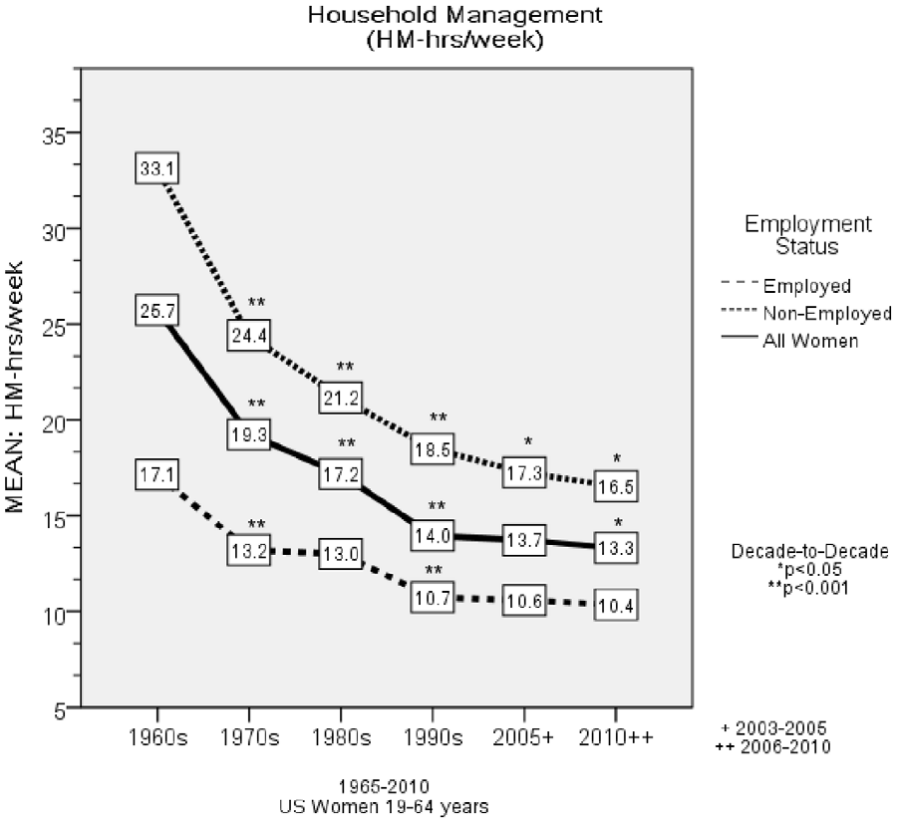
Household Management Hours per Week by Employment Status. This figure depicts the decade to decade changes in Household Management Hours per Week (HM-hr/week) for all women and by employment status.

**Figure 2:**
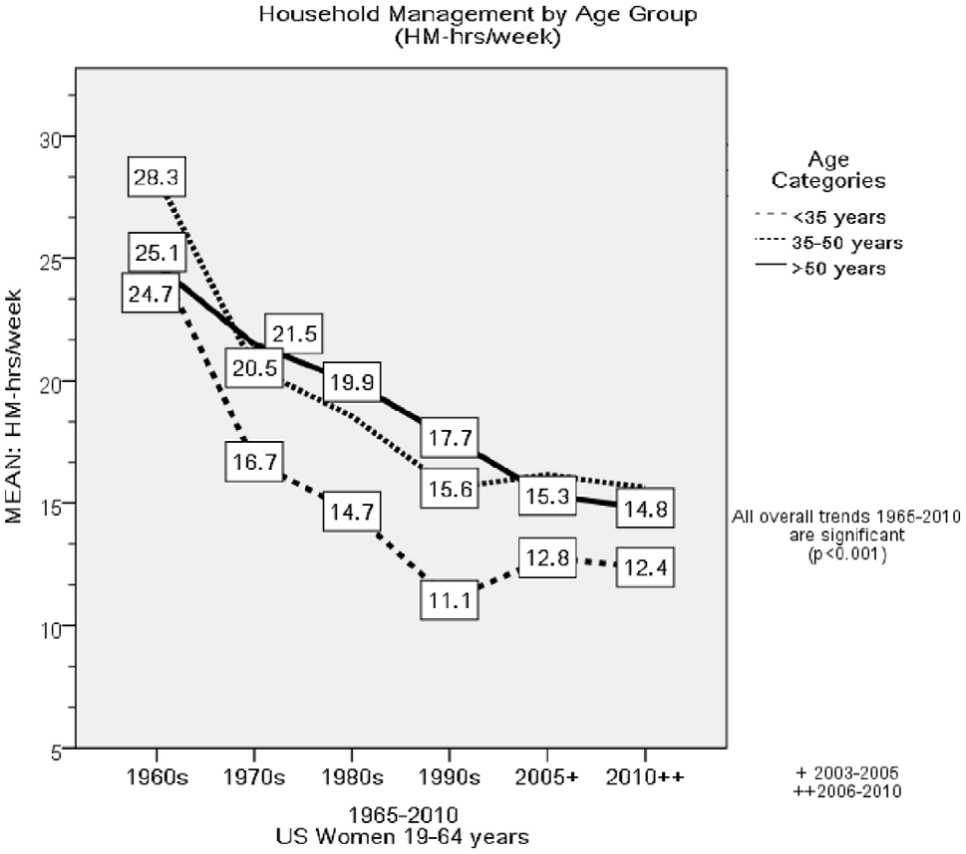
Household Management Hours per Week by Age Group. This figure depicts the decade to decade trends in the change in Household Management Hours per Week (HM-hr/week) by age group.

From 1965 to 2010, there were significant trends for decrements in HM-hr/week (*F* = 730.6, *p*<0.001) for women 19–64 years old (*figures 1* *&* *2* *)* and an overall decline of 12.4 hr/week (*F* = 153.9, p<0.001), with non-employed women demonstrating a 16.6 hr/week decline (*F* = 152.4, *p*<0.001*)* and employed women a 6.8 hr/week decline in HM-hr/week (*F* = 32.5, *p*<0.001). Figure 2 depicts the trends in HM-hr/week by age group. There were significant trends for decrements in HM-hr/week in all age groups from 1965–2010 (p<0.001).

### Household Management Energy Expenditure (HMEE)

**Figure 3:**
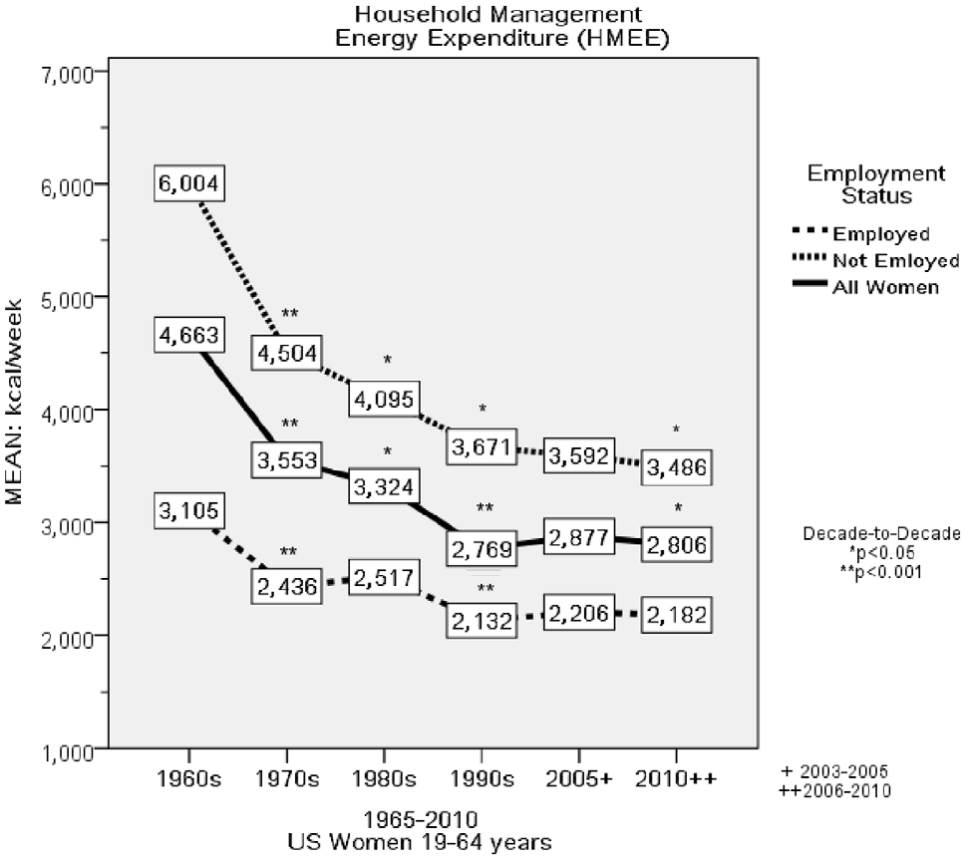
Household Management Energy Expenditure per Week. This figure depicts the decade to decade change in Household Management Energy Expenditure per Week (HMEE/week) for all women and by employment status.

There was a significant trend for decrements in HMEE from 1965 to 2010 (*F* = 458.2, p<0.001), and significant decade-to-decade declines in HMEE from 19.5 Mj/week, 2.8 Mj/day (4663 kcal/week; 666 kcal/day) in 1965 to 11.8 Mj/week, 1.7 Mj/day (2806 kcal/week; 400 kcal/day) in 2010 (*F* = 93.1, *p*<0.001). This was a decrease of 7.7 Mj/week, 1.1 Mj/day (1857 kcal/week; 265 kcal/day) over the 45-year period (*figure 3* *)*.

Non-employed women experienced the greatest decline in HMEE, from 25.1 Mj/week or 3.6 Mj/day (6004 kcal/week; 857 kcal/day) in 1965 to 14.6 Mj/week, 2.1 Mj/day (3486 kcal/week; 498 kcal/day) in 2010 (*F* = 94.9, p<.001), a 42% decrement in EE of 10.5 Mj/week, 1.5 Mj/day (2518 kcal/week; 360 kcal/day). Employed women exhibited a smaller decrement of 3.9 Mj/week, 0.6 Mj/day (923 kcal/week; 132 kcal/day), from 13.0 Mj/week, 1.9 Mj/day (3105 kcal/week, 444 kcal/day) in 1965 to 9.1 Mj/week, 1.3 Mj/day (2182 kcal/week; 311 kcal/day) in 2010 (*F* = 15.8, p<0.001).

### Activity Displacement

#### Screen-based media use (TV and computer)

**Figure 4:**
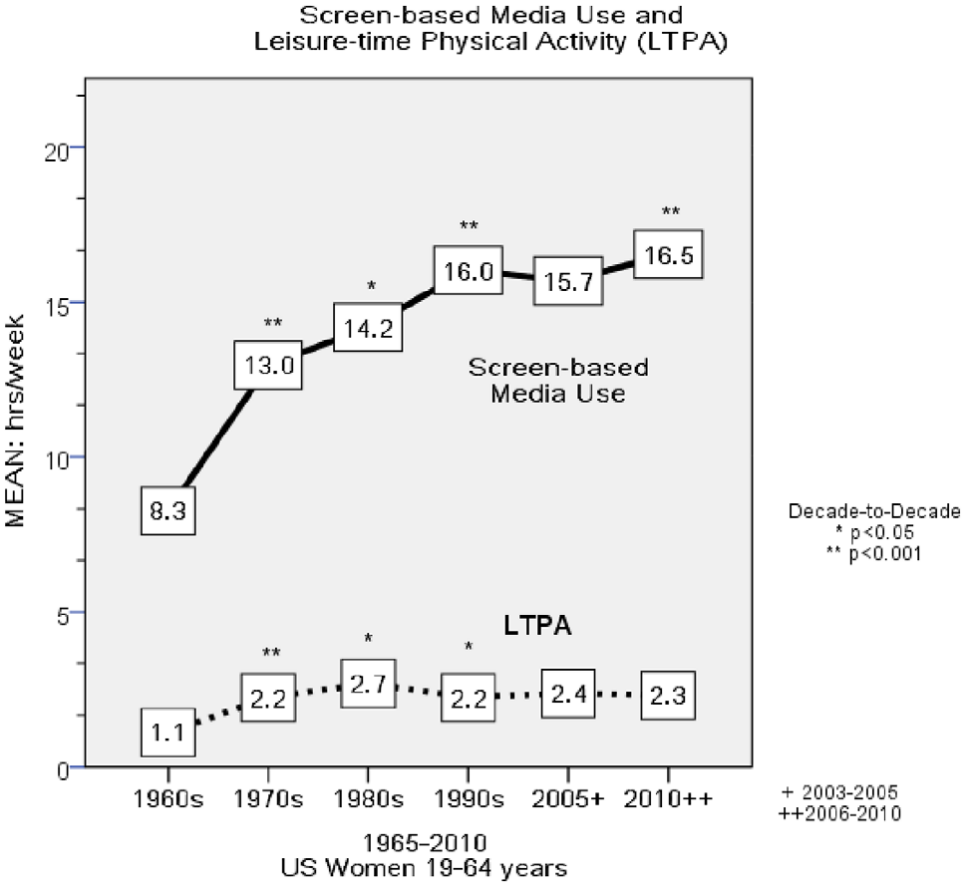
Screen-based Media Use & Leisure-Time Physical Activity. This figure depicts the decade to decade changes in Screen-based media use and Leisure-Time Physical Activity (LTPA) for all women.

From 1965 to 2010, there was a significant trend for increments in screen-based media use in all women (*F = *600.7, p<0.001) (*figure 4* *)*. In 1965, women reported 8.3 hr/week in screen-based media, this value increased to 16.5 hr/week in 2010 (*F = *130.7, p<0.001). All decade-to-decade transitions were significant with the exception of 1990s–2005. Screen-based media time for non-employed women increased from 10.0 hr/week in 1965 to 19.6 hr/week in 2010 (*F* = 91.2, *p*<0.001). Screen-based media time for employed women increased from 6.2 hr/week in 1965 to 13.7 hr/week in 2010 (*F* = 57.8, *p*<0.001).

#### Leisure-time Physical Activity (LTPA)

Women increased their self-reported LTPA from 1.1 hr/week in 1965 to 2.3 hr/week in 2010 (*F = *26.6, *p*<0.001), an increase of 1.2 hr/week (10.3 minutes per day). The 1960s–1970s and the 1970s–1980s transitions were significant exhibiting increases of 1.1 hr/week (*F = *47.7, *p*<001) and.44 hr/week (*F = *4.9, p<0.05). Reported LTPA decreased from 1980 to 1990 by.45 hr/week (*F* = 6.0, *p*<0.05) with no significant change from 1990–2010. Non-employed women increased their LTPA from 1.2 hr/week in 1965 to 2.5 hr/week in 2010 (*F = *38.4, *p*<.001), an increase of 1.3 hr/week (11.1 minutes/day). Employed women increased from 1.0 to 2.2 hr/week (*F = *18.8, *p*<0.001), an increase of 1.2 hr/week (10.3 minutes/day).

#### Leisure-time Physical Activity Energy Expenditure (LTPAEE)

Women increased LTPAEE by 1.9 Mj/week (460 kcal/week) from 1965 to 2010 (*F* = 42.7, *p*<0.001). In 1960s, LTPAEE was 1.4 Mj/week (324 kcal/week) and increased to 2.8 Mj/week (667 kcal/week) in 1970s (*p*<0.001) and 3.5 Mj/week (839 kcal/week) in 1980s (*p*<0.05). LTPAEE decreased to 3.0 Mj/week (716 kcal/week) in 1990s (*p*<0.05) but increased to 3.3 Mj/week (796 kcal/week) in 2005 (*p*<0.05). There was a non-significant change from 3.3 Mj to 3.2 Mj per week (796 to 783 kcal/week) from 2005–2010 (*p = *4.94).

## Discussion

From 1965 to 2010, there was a large and significant decline in the time women allocated to HM (*figure 1* *)*. By the 1990s, women spent more time in screen-based media use (e.g., watching TV) than in cooking, cleaning, laundry and LTPA combined. A major consequence of the 12 hr/week decline in HM was a considerable decrement in HMEE, with non-employed women experiencing the largest decrease (10.5 Mj/week, 1.5 Mj/day; 2518 kcal/week, 360 kcal/day; *figure 3*). In parallel with the considerable decline in HMEE was a substantial increase in the amount of time spent in screen-based media use (8.3 hr/week), and a smaller but statistically significant increase in LTPA (1.2 hr/week) (*figure 4*).

Technology has played a large part in the decline in HM hr/week [35], [44], [45]. As automation improved the efficiency and decreased the requisite exertion of household tasks, the greatest decrements in HMEE were in individuals who previously allocated the greatest amount of time to HM (e.g., “stay-at-home” moms). Advances in food manufacturing led to an increase in the use of prepackaged, microwaveable meals and a consequent decrease in the time spent preparing food [46], [47]. In 1970, less than 1% of all homes had a microwave oven and <20% a dishwasher; by 2005, more than 90% of homes had a microwave and >60% a dishwasher. Post-meal clean-up was minimized (via disposable food containers) and mechanized (via the dishwasher) so individuals had the freedom to perform other activities (e.g., watch TV). Another development relevant to food preparation was the increased reliance on the food service industry. In 2000, nearly 50% of all food costs were spent on food away from home, compared with <30% in 1965, despite the decreasing relative costs of restaurant foods [46], [48].

One of the most dominant factors in the decrement of HMEE over time appears to be the change in women”s social roles. Early in the 20^th^ century, women allocated the vast majority of their time to unpaid HM activities. Beginning in the 1950s, women began to divide their time between unpaid HM activities and paid employment [49]. From 1950 to 2000, women”s full-time employment increased from 34% to 60% [50] and the full-time employment of mothers with children increased from 19 to 57 percent [36], [51]. This demographic transition lead to a decrease in the time women allocated to HM, child care, and personal care [31], [35], and dramatically decreased the amount of time that working women with children allocated to HM. Mothers who are employed full-time perform less childcare (8 hr/week), less housework (10 hrs/week) and achieve less sleep (∼3 hr/week) than non-employed or part-time working mothers. [35], [36], [52] This shift from more active (i.e., energetically costly) activities such as HM to more sedentary occupational activities has an obvious impact of energy expenditure and health. [9], [30], [53] The resulting decrease in HM and HMEE was only partially offset by the increases in LTPA.

### Energy Balance, Obesity and Health

Given the complexity of human behavior and the behavioral and physiological compensatory mechanisms engendered by alterations in energy balance, there are no models that allow valid extrapolations from decreases in HMEE to increments in population-level weight-gain. Nevertheless, the declines in HM-hr/week and HMEE parallel the increments in screen-based media use suggesting that as the overall time and energy spent on PA decreased, the amount of time spent in sedentary behaviors increased.

### Intergenerational Effects of Inactivity

Given the intergenerational effects of inactivity on metabolic function, health and obesity [54], [55], [56], the dramatic decreases in HM and HMEE may result in ever-increasing increments in obesity and NCDs in subsequent generations. Recent research has demonstrated that a majority of pregnant women spend more than 50% of their waking hours in sedentary behavior and that >15% of pregnant women have reported spending more than 5 hr/day in screen-based media use [57], [58]. The trend of the reallocation of time from active behaviors such as HM to sedentary activities has obvious and significant health consequences for future generations [9], [53].

### Population Trends and Obesity

The confluence of our results and research on other PA domains (e.g., transport [59], occupational [30], housework [44], sedentary behavior [60]) suggests a considerable decrement in total PA and substantial increases in sedentary behavior during the past few decades. This growing body of research suggests that the rise in bodyweight and obesity may be due to decreases in PA alone. In fact, these data from multiple PA domains suggest that the decrement in EE may be so large that current levels of obesity would be significantly higher if compensatory responses (e.g., decreased EI or increased LTPA) were absent.

### Strengths and Limitations

#### Strengths

This study represents the first detailed analyses of trends in HMEE derived from nationally representative historical time-use databases. HMEE was derived using an empirically supported protocol for the translation of PA into EE [39], [61]. Our results on the allocation of time to HM, LTPA and screen-based media use are in agreement with other research [33], [52], [62], [63]. Furthermore, we used a conservative estimate of the mean intensity of household activities (i.e., 2.8 METs) based on the convention established by the FAO/WHO/UNU in 1985 [37] since the available data did not have the level of detail necessary to calculate EE for each specific component of HM. Importantly, the use of this value facilitates future examinations of HMEE across developed nations. Moreover, our results on activity displacement (i.e., the reallocation of time away from HM to sedentary pursuits) are bolstered by investigations independent of the AHTUS datasets demonstrating decreased HM [64], and Nielsen research that suggests that TV viewing has increased at a rate greater than indicated in our findings [65], [66].

#### Limitations

The two most significant limitations are the use of harmonized datasets and self-reported data. The harmonization process of the datasets is complex and was not performed by our research group. Nevertheless, the process is quite robust and there are hundreds of peer-reviewed publications and academic texts that used these harmonized datasets (see http://www.timeuse.org).

The biases associated with self-reported data are not trivial and limit our results; especially biases induced via social desirability and the cognitive demands of recording the allocation of time. Nonetheless, these biases may be omnipresent and stable, and therefore may not present a significant threat to our analysis of trends. For example, TV viewing was not considered a socially desirable behavior throughout the period of study [66], [67]; as such, the self-reported increments in screen-based media use over five decades lend support to our contention that longitudinal trends may be less affected than analyses of static cross-sectional data.

Additionally, our analyses accounted only for the time allocated to HM and not how technological advances (e.g., self-propelled vacuum cleaners, food processors) have altered the effort (i.e., intensity) of HM. Accelerometry and indirect calorimetry research [68], [69], [70] suggest higher estimates for specific components of HM activities than either the 2011 Compendium [39] or FAO/WHO/UNU report [37]. Nevertheless, this limitation does not diminish our estimates of the decrement in HM hr/week and suggests a modest underestimation of HMEE that is supportive of our overall interpretation of decreased PA and HMEE.

### Implications for Public Health Policy

The estimated reductions in HM (>12 hr/week) and HMEE (>7.7 Mj/week; 1850 kcal/week) were not compensated by the observed increases in LTPA (<1.3 hr/week; 1.9 Mj/week; 460 kcal/week); nor can the decrements be adequately compensated by meeting the 2008 federal PA recommendations of 2.5 hr/week of moderate intensity PA (MPA) (∼2.3 Mj/week; ∼560 kcal/week) [71] or the Institute of Medicine”s recommendation for the maintenance of a healthy weight of 60 minutes/day of MPA (∼6.6 Mj/week; 1575 kcal/week) [72]. Future PA recommendations may need to be increased to overcome the total decrement in the various domains of PA (e.g., transport [59], occupational [30]) that some sub-groups (e.g., stay-at-home moms) have experienced over the past half century.

### Conclusion

Physical inactivity is one of the leading causes of morbidity and mortality in the world [2], [73], [74] and yet is all too often under-emphasized in clinical, educational and public health settings [75], [76], [77]. While the attraction of simple causation in the etiology of obesity is powerful (e.g., economic forces cause obesity), the development of effective strategies and tactics to ameliorate the effects of NCDs and obesity necessitates a broad understanding of the complexities of human behavior and energy metabolism, inclusive of EI, EE and PA.
